# Adsorption of Cr(VI) in Aqueous Solution Using a Surfactant-Modified Bentonite

**DOI:** 10.1155/2020/3628163

**Published:** 2020-03-21

**Authors:** Johnatan D. Castro-Castro, Iván F. Macías-Quiroga, Gloria I. Giraldo-Gómez, Nancy R. Sanabria-González

**Affiliations:** ^1^Department of Physics and Chemistry, Universidad Nacional de Colombia Sede Manizales, Campus La Nubia, km 7 Vía al Aeropuerto, AA 127, Manizales, Colombia; ^2^Department of Chemical Engineering, Universidad Nacional de Colombia Sede Manizales, Campus La Nubia, km 7 Vía al Aeropuerto, AA 127, Manizales, Colombia

## Abstract

Clay minerals can be modified organically by a cationic surfactant resulting in materials known as organoclays. The organoclays have been used as adsorbents of most of the organic contaminants in the aqueous solution and oxyanions of the heavy metal. In this study, a Colombian bentonite was modified with hexadecyltrimethylammonium bromide to obtain an organobentonite, and its capacity to adsorb Cr(VI) oxyanions in the aqueous solution was evaluated. The effect of pH, stirring speed, adsorbent amount, contact time, and ionic strength were investigated at 25°C. Stirring speeds above 200 rpm, contact times greater than 120 min, and the addition of NaCl (0.1 to 2.0 mM) did not have a significant effect on Cr(VI) removal. The influence of the adsorbent amount and pH on Cr(VI) adsorption was studied by the response surface methodology (RSM) approach based on a complete factorial design 3^2^. Results proved that the Cr(VI) adsorption follows a quadratic model with high values of coefficient of determination (*R*^2^ = 95.1% and adjusted *R*^2^ = 93.9%). The optimal conditions for removal of Cr(VI) from an aqueous solution of 50 mg/L were pH of 3.4 and 0.44 g amount of the adsorbent. The adsorption isotherm data were fitted to the Langmuir and Freundlich adsorption isotherm models, and the model parameters were evaluated. The maximum adsorption capacity of Cr(VI) onto organobentonite calculated from the Langmuir model equation was 10.04 ± 0.34 mg/g at 25°C. The results suggest that organobentonite is an effective adsorbent for Cr(VI) removal, with the advantage of being a low-cost material.

## 1. Introduction

The removal of toxic substances from water and wastewater has been a core interest of many scientists and researchers around the globe over the past decades [1]. The rapid growth of industrialization and urbanization has resulted in increased environmental pollution, which has been a serious cause of public concern [2]. Among these pollutants, contribution of heavy metals is a major concern because of their toxicity, bioaccumulation, persistence, and nonbiodegradable nature [3]. Heavy metals such as cadmium, zinc, lead, chromium, nickel, copper, vanadium, platinum, and silver are generated in electroplating, electrolysis depositions, conversion-coating, and anodizing-cleaning, milling, and etching industries [1, 4].

Chromium is used in industries such as metallurgy, electroplating, production of paints and pigments, tanning, wood preservation, and pulp and paper production. The chromium pollution associated with these industries has adverse effects on aquatic ecosystems [5]. Chromium is ranked as number 17 on the priority list of hazardous substances according to the agency for Toxic Substances and Disease Registry of the United States [6].

Heavy metal removal from the effluents can be achieved by physicochemical treatment processes such as ion exchange, coagulation-flocculation, chemical precipitation, evaporation, membrane filtration, electrochemical treatments, and adsorption [1, 4]. Adsorption process has attracted attention of many researchers because of its low cost, ease of operation, design flexibility, and high efficiency for removal of inorganic and organic pollutants from wastewater [1, 4, 7].

Clay minerals and their modified derivatives are a family of materials which can be used for the adsorption of most of the chemical contaminants from the aqueous solution [7]. Among this family of adsorbents, those based on montmorillonite have been used as adsorbents for cationic contaminants, including heavy metal cations [1] and cationic dyes [8]. Montmorillonite is a phyllosilicate that belongs to the group of smectites, and it is the main component of bentonite clay. The hydrophilic nature of bentonite makes this material an ineffective adsorbent of organic compounds [9] and chromate oxyanions [10]. In the interlayer space of the sodium bentonite, there are ions of compensation Na^+^, which cannot be exchanged by the Cr(VI) species because the chromium in the aqueous solution exists as HCrO_4_^−^ and CrO_4_^2−^, depending on the pH of the solution [11].

Smectite clay minerals can change their naturally hydrophilic character into organophilic, acting as adsorbents for organic compounds and inorganic anions. The organophilization consists of cationic surfactant (quaternary ammonium salts) addition with a chain of twelve or more carbon atoms to aqueous dispersions at an inorganic cation (typically Na^+^) exchange by alkylammonium cations [7]. Intercalation of a larger size of the surfactant cations into layers of clay minerals not only changes the surface properties from hydrophilic to hydrophobic, but also greatly increases the interlamellar distance (basal spacing) of the layers, thus easing the attraction of organic molecules [9] and chromate anions [10, 12].

The adsorption of Cr(VI) on organoclays has been previously investigated [12–15]; however, the optimal conditions of the adsorption process of Cr(VI) in organobentonite have not been studied by the multivariate experimental design. In this study, the effect of the variables involved in the adsorption process of Cr(VI) on the organobentonite (pH, stirring speed, amount of adsorbent, contact time, and ionic strength) was evaluated. The response surface methodology (RSM) was used to analyze the effect of pH and adsorbent amount on the Cr(VI) adsorption efficiency by organobentonite and establish the optimal conditions of the adsorption process. Additionally, batch experiments are performed to obtain the Cr(VI) adsorption capacity at different temperatures.

## 2. Experimental

### 2.1. Reagents and Materials

The surfactant hexadecyltrimethylammonium bromide (HDTMA-Br, C_19_H_42_BrN, >98.0% purity) was purchased from Panreac®. The clay used was a Colombian bentonite sample from Armero-Guayabal (Tolima). The main clay mineral of the bentonite used in this study is the montmorillonite (48 wt%), and its mineralogical and chemical composition has been previously reported [16].

A stock solution containing 1000 mg of Cr(VI) per liter was prepared by dissolving 0.1 N K_2_Cr_2_O_7_ (Titrisol, Merck KGaA, Darmstadt, Germany) in double-distilled water and was used to prepare the solutions by appropriate dilution. The pH of the aqueous solution of Cr(VI) as prepared was 4.6.

### 2.2. Synthesis of Surfactant-Modified Bentonite

The clay fraction (<2 *μ*m) was obtained by gravitational sedimentation of the clay sample. Sodium bentonite (denoted as Na-Bent) was prepared from the clay fraction using a 5% suspension of clay in 1 M NaCl solution. The suspension was agitated for 24 h at room temperature, and the solution was separated from the clay by centrifugation at 10000 rpm. This procedure was repeated three times, and the homoionic Na-bentonite was repeatedly washed with distilled water until a negative test for chloride ions in the washing was obtained [17]. The Na-Bent was dried in an over for 72 h at 60°C, and then ground and sieved in a 100-mesh screen.

The organobentonite was prepared by cationic exchange between the bentonite homoionized with sodium (CEC of the Na-Bent of 63.02 meq/100 g) and a cationic organic surfactant, hexadecyltrimethylammonium bromide (HDTMA-Br). The proportion of the surfactant used in the synthesis of organobentonite was 1.5 times the proportion of cation exchange capacity of sodium bentonite, a value recommended in literature [18], with a procedure similar to that used in a previous study [19]. For this, 20 g of Na-Bent was added to 500 mL of distilled water, and the suspension was stirred for 12 h to generate the swelling of the bentonite, and then the suspension was added with 7.03 g of HDTMA-Br (dissolved in 100 mL of distilled water) and the mixture was stirred for 24 h. The organobentonite (denoted as HDTMA-Bent) was separated by centrifugation and washed repeatedly until a negative bromide test was obtained with 0.1 M of AgNO_3_. Finally, the HDTMA-Bent was dried at 80°C for 24 h and at 100°C for 2 h and then ground and sieved in 100-mesh screen. Na-Bent and HDTMA-Bent were characterized by X-ray diffraction (XRD), Fourier transform infrared spectroscopy (FT-IR), and nitrogen adsorption at 77 K. The diffraction patterns were taken to a LabX Shimadzu XRD-6000 diffractometer with Cu K*α* radiation (steps of 0.02° 2*θ* and 2 s/step). Fourier transform infrared spectrometry (FT-IR) was recorded from samples pressed into pellets with KBr powder by using a Nicolet iS5 (Termo Scientific). Nitrogen adsorption-desorption isotherms were determined in a Micromeritics ASAP 2020 instrument at 77 K after outgassing the samples for 3 h at 90°C, followed by 2 h at 120°C in a vacuum.

### 2.3. Adsorption Experiments

Batch adsorption tests were carried out in 100 mL glass Erlenmeyer with 50 mL of the Cr(VI) solution of 50 mg/L and placed in a thermostated bath at 25°C with magnetic stirring. The initial methodological design to evaluate the adsorption of Cr(VI) was proposed with a unique approach, in which the effect of each factor (pH, stirring speed, amount of adsorbent, contact time, and ionic strength) was analyzed independently, maintaining the other constants according to the experimental conditions established in Table 1. The pH of the solution was adjusted with 0.1 M HCl and 0.1 M NaOH solutions, and the effect of ionic strength on the adsorption of Cr(VI) was carried out varying the concentrations of the NaCl solution.

The adsorbent was separated by centrifugation at 5000 rpm for 5 min, and the supernatant solution was filtered in a 0.22 *μ*m membrane and then carefully transferred to glass flasks to determine the concentrations of chromium present in the aqueous medium. All the tests were performed in triplicate. The concentrations of the chromium were measured by flame atomic absorption spectrometry (iCE 3500, Thermo Scientific, Germany), with an air-acetylene flame. The removal efficiency onto the organobentonite was calculated using the following equation:

(1) $$\begin{array}{c} \text{Cr}\left(\text{VI}\right)\text{ removal }\left(\%\right)=\frac{C_{i}-C_{t}}{C_{i}}\times 100\;, \end{array}$$

where *C*_*i*_ and *C*_*t*_ (mg/L) are the liquid-phase concentrations of the Cr(VI) at initial and time *t*, respectively.

### 2.4. Experimental Design and Adsorption Isotherm

The influence of the adsorbent amount and pH on Cr(VI) adsorption was studied by the response surface methodology (RSM) approach based on the complete factorial design 3^2^. The amount of Cr(VI) adsorbed at 25, 30, and 35°C was evaluated at different initial concentrations of Cr(VI) at the optimal conditions obtained from the experimental design. The equilibrium adsorption capacity (*q*_*e*_) was determined by the following equation:

(2) $$\begin{array}{c} q_{e}=\frac{\left(C_{i}-C_{e}\right)}{W}\times V, \end{array}$$

where *C*_*i*_ and *C*_*e*_ are the initial and equilibrium concentrations of the Cr(VI) in the liquid phase (mg/L), *V* is the aqueous phase volume (L), and *W* is the mass (g) of the adsorbent.

## 3. Results and Discussion

### 3.1. Characterization of the Clay and Organobentonite

**Figure 1:**
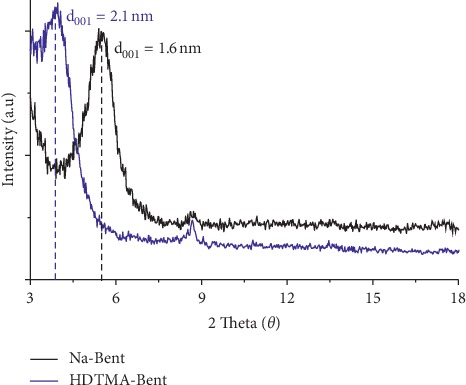
XRD patterns of sodium bentonite (Na-Bent) and organobentonite (HDTMA-Bent).

Figure 1 shows the diffraction patterns of sodium bentonite and organobentonite. The modification of the bentonite with the quaternary ammonium salt caused an increase in the basal spacing (d_001_) of the layers from 1.6 nm to 2.1 nm brought about by HDTMA^+^ cation intercalation. Macías-Quiroga et al. [16] and Otavo-Loaiza et al. [19] have reported similar basal spacing values for this bentonite and the bentonite modified with HDTMA-Br. The increase in the interlayer spacing indicates that the HDTMA^+^ cations intercalated in the interlamellar space were arranged in the form of a pseudo-trimolecular layer [20].

To determine the specific surface area of bentonite and organobentonite, a model BET was used. External surface area and porous volume were assessed by *t* curves using the Harkins–Jura equation [21]. The specific surface area and pore volume of the bentonite were reduced from 59.9 to 2.5 m^2^/g and from 0.082 to 0.008 cm^3^/g, respectively, when the bentonite was modified with the surfactant (HDTMA-Bent). Similar results have been reported by Jacobo-Azuara et al. [22] when bentonite was modified with hexadecyltrimethylammonium bromide.

**Figure 2:**
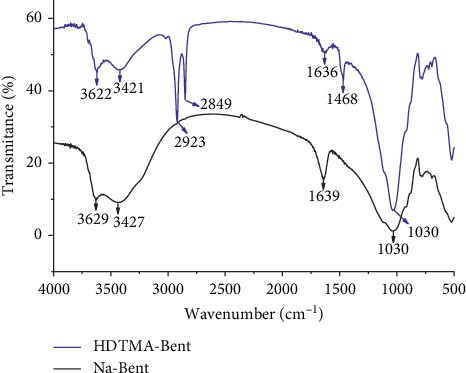
FT-IR spectra of sodium bentonite (Na-Bent) and organobentonite (HDTMA-Bent).

Figure 2 shows the FT-IR spectra of Na-Bent and HDTMA-Bent. The two spectra show the presence of –OH as stretching bands at 3622–3629 cm^−1^. The large band at 1030 cm^−1^ corresponds to the Si-O stretching vibration. The above signals can be considered characteristic of dioctahedral smectite [16, 23]. Broad bands centered near 3421–3427 cm^−1^ and 1636–1639 cm^−1^ are due to the–OH stretching mode of the interlayer water [24]. Adsorption bands of pure HDTMA at 2923 cm^−1^ and 2849 cm^−1^ correspond to the CH_2_ asymmetric stretch modes and CH_3_ symmetric stretch modes, respectively [25]. The band at 1468 cm^−1^ is assigned mainly to N-C stretching of the cationic surfactant [9].

### 3.2. Adsorption Study

#### 3.2.1. Effect of pH

**Figure 3:**
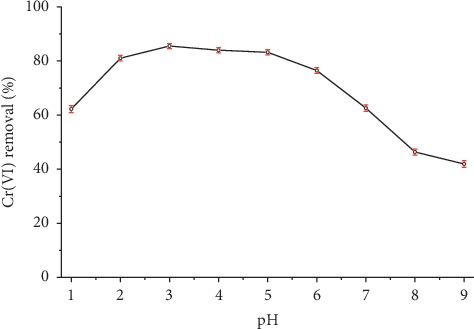
Effect of pH on removal of Cr(VI) using organobentonite. Conditions: *C*_*i*_  = 50 mg/L, *V* = 50 mL, *T* = 20°C, stirring speed = 200 rpm, HDTMA-Bent = 0.2 g, and contact time = 60 min.

The removal of Cr(VI) using organobentonite was highly dependent on the pH of the solution, as shown in Figure 3. The hexavalent chromium ions can exist as hydrogen chromate (HCrO_4_^−^), chromate (CrO_4_^2−^), or dichromate (Cr_2_O_7_^2−^), depending upon the pH of the solution [26]. At pH 3, the maximum removal by adsorption was found, and when the pH increased from 5 to 9, the removal of Cr(VI) decreased drastically. According to the Cr(VI) speciation diagram, the HCrO_4_^−^ is the predominant specie in the pH interval 2–4, HCrO_4_^−^ and Cr_2_O_7_^2−^ ions are common at pH interval 2–6, and CrO_4_^2−^ is the predominant specie in the pH range 9–12 [11]. The affinities of HCrO_4_^−^, Cr_2_O_7_^2−^, and CrO_4_^2−^ ions to the modified bentonite are different, with the preference of the first two species in acid solutions [15].

Using SWAXS analysis (small-angle angle X-ray scattering) pattern recorded before and after adsorption of chromium, Majdan et al. [15] proposed the formation of alkylammonium chromates: (HDTMA) (HCrO_4_), (HDTMA)_2_Cr_2_O_7,_ and to the lesser extent (HDTMA)_2_CrO_4_ in the interlayer space of the bentonite. The removal of the Cr(VI) occurs by electrostatic interaction between the species HCrO_4_^−^ and Cr_2_O_7_^2−^ with the surface of the positively charged organobentonite and with the alkylammonium cations located in the interlayer space [15]. In general, the highest adsorption of Cr(VI) occurred at pH between 3 and 4, with removals greater than 83.97 ± 0.97%, and this performance has been linked to the predominant adsorption of HCrO_4_^−^ specie [27].

#### 3.2.2. Effect of Stirring Speed

**Figure 4:**
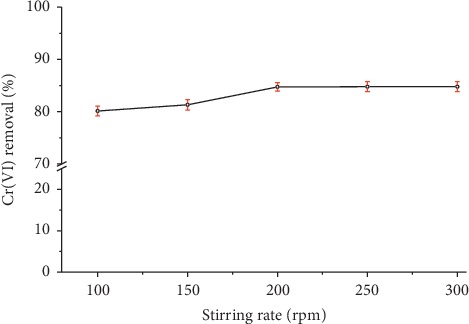
Effect of stirring speed on removal of Cr(VI) using organobentonite. Conditions: *C*_*i*_ = 50 mg/L, *V* = 50 mL, *T* = 20°C, pH = 3.0, HDTMA-Bent = 0.2 g, and contact time = 60 min.

The stirring speed is an important parameter in the adsorption process since it influences the distribution of the solute in the bulk solution and the formation of the external boundary film [28]. The effect of the stirring speed on the removal of Cr(VI) onto organobentonite evaluated for a contact time of 1 h is shown in Figure 4. At 200, 250, and 300 rpm, the removal of Cr(VI) tended at a constant value, around 84.78 ± 0.95%. At low stirring speeds (100 and 150 rpm), slightly lower removals of Cr(VI) were obtained, which demonstrate that increasing the stirring speed (≥200 rpm) increases the turbulence in the mixing zone [29].

The removal of Cr (VI) was not positively correlated with the stirring speed between 200 and 300 rpm because the contact time of 1 h guaranteed equilibrium time conditions in the adsorption tests. It has been established that the stirring speed affects the equilibrium time, but the equilibrium adsorption capacity is not a function of the stirring speed [28]. The stirring speed increase causes an increase in the Reynolds number, in the energy dissipation, and in the turbulence in the mixing zone.

Several studies on adsorption of Cr(VI) in modified minerals do not include data of stirring speed [30] and others only specify the value used. For example, Thanos et al. [31] carried out all the experiments in batch reactors under continuous stirring at 600 rpm, and Hommaid and Hamdo [32] and Slimani et al. [33] report that the suspension was stirred at 150 rpm on a shaker and in a magnetic stirrer, respectively.

#### 3.2.3. Effect of Amount of Adsorbent

**Figure 5:**
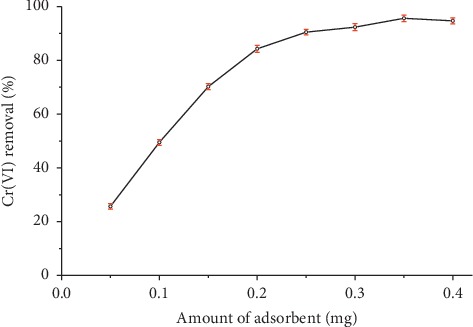
Effect of amount adsorbent on the removal of Cr(VI) using organobentonite. Conditions: *C*_*i*_  = 50 mg/L, *V* = 50 mL, *T* = 20°C, pH = 3.0, stirring speed = 200 rpm, and contact time = 60 min.

Figure 5 shows the effect of amount of organobentonite on Cr(VI) adsorption. When the amount adsorbent step from 0.05 to 0.4 g of Cr(VI) removal increase from 25.65 ± 1.01 to 94.69 ± 1.32%, it was observed that the adsorption of the Cr(VI) increased rapidly with increasing amount of adsorbent from 0.05 to 0.20 g and slightly enhanced from 0.25 to 0.40 g. These results can be attributed to the increased adsorbent surface area and availability of more adsorption sites. However, it has been established that a higher availability of adsorption sites results in less effective utilization of the sites by the chromate ions due to saturation deficiency [31].

#### 3.2.4. Effect of Contact Time

**Figure 6:**
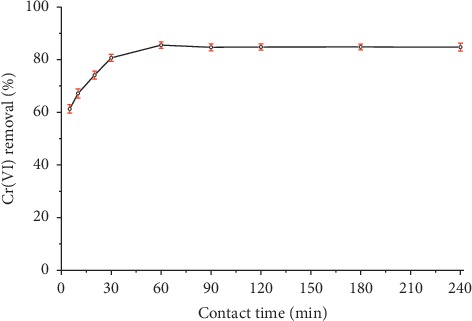
Effect of contact time on removal of Cr(VI) using organobentonite. Conditions: *C*_*i*_  = 50 mg/L, *V* = 50 mL, *T* = 20°C, pH = 3.0, stirring speed = 200 rpm, and HDTMA-Bent = 0.2 g.

The contact time is one of the most important parameters for the industrial application of an adsorbent. A good adsorbent should not only provide high adsorption capacities, but also produce a fast process [28]. The influence of contact time on the Cr(VI) adsorption on organobentonite is shown in Figure 6. The Cr(VI) removal on organobentonite improved rapidly by the increase in contact time from 5 to 30 min. Between 30 and 60 min, the Cr(VI) removal increased slightly, and after 90 min, the adsorption capacity became constant and the adsorption achieved equilibrium.

Slimani et al. [33] evaluated the kinetic and thermodynamic parameters of chromium adsorption on a bentonite modified with HDTMA-Br and found that the adsorption of chromium was very fast at the beginning and then approached equilibrium after 15 min. Similar results were obtained by Thanos et al. [31] finding that chromate ion adsorption occurred at a very high stirring at the initial stages (*t* = 0–10 min) for four HDTMA-Br-modified minerals. Although it has been established that the removal of Cr(VI) using organoclay as an adsorbent occurs very rapidly, some researchers have employed high equilibrium times. Jacobo-Azuara et al. [22] manually shaked the solution for four to five times daily and found that 5 days are sufficient to reach equilibrium.

#### 3.2.5. Effect of Strength Ionic

**Figure 7:**
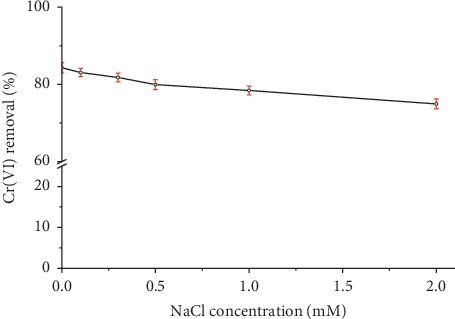
Effect of ionic strength on removal of Cr(VI) using organobentonite. Conditions: *C*_*i*_  = 50 mg/L, *V* = 50 mL, *T* = 20°C, pH = 3.0, stirring speed = 200 rpm, HDTMA-Bent = 0.2 g, and contact time = 60 min.

Wastewaters from different industries contain various types of salts, and their presence leads to high ionic strength, which may significantly affect the performance of the adsorption process [34]. The effect of the addition of NaCl on the Cr(VI) removal is shown in Figure 7. The Cr(VI) adsorption on organobentonite decreased slightly when increasing the concentrations of NaCl in the solution from 0.1 to 2.0 mM. The Cr(VI) solution of 50 mg/L was prepared by dissolving 0.1 N K_2_Cr_2_O_7_ (Titrisol, Merck KGaA) in double-distilled water, and for the ionic strength tests, the pH of the solution was adjusted to 3.0, adding 0.1 M HCl. Therefore, the solution used in the adsorption tests contains H^+^, Cl^−^, K^+^, HCrO_4_^−^, and CrO_4_^2−^ ions, which generate ionic strength. An increase in ionic strength generates the compression of the diffuse double layer on the adsorbent and affects the adsorption process [34].

Gładysz-Płaska et al. [35] studied the influence of ionic strength on Cr(VI) adsorption on HDTMA-clay, finding that an increase in the ionic strength of the solution resulted in a decrease in Cr(VI) adsorption. Under the test conditions (*T* = 293 K, pH = 5.8, 0.4 of HDTMA-clay, equilibrium time = 6 h, and 0.1 mmol/L NaCl), chloride ions reduced Cr(VI) adsorption from 59 to 25%.

In conclusion, the main variables that affected the adsorption of Cr(VI) on the organobentonite were the amount of adsorbent and the pH. Stirring speed above 200 rpm and contact time exceeding 90 min did not have a significant effect on Cr(VI) removal. As the solution used in the adsorption tests contains H^+^, Cl^−^, K^+^, HCrO_4_^−^, and CrO_4_^2−^ ions, which generate ionic strength, in the subsequent tests, the addition of NaCl to the Cr(VI) aqueous solution was not considered.

### 3.3. Experimental Design

The effects of amount of adsorbent (A) and the pH (B) were analyzed using response surface methodology with a complete factorial design 3^2^. The levels for A and B were 0.3, 0.4, and 0.5 g and 2.0, 3.0, and 4.0, respectively. The other variables, temperature, contact time, and stirring speed were kept constant at 25°C, 150 min, and 230 rpm, respectively. Design Expert version 9.0.6.2 (StatEase, Inc., Minneapolis, USA) was used for regression analysis of the obtained experimental data. In the optimization process, the response variable can be related to chosen variables by linear or quadratic models [36]. A quadratic model is given in the following equation:

(3) $$\begin{array}{c} Y=\;\beta_{0}+\sum_{i=1}^{k}\beta_{i}X_{i}+\sum_{i=1}^{k}\beta_{ii}X_{i}^{2}\sum_{i=1}^{k-1}\sum_{\begin{array}{c} j=2\\ j>i \end{array}}^{k}\beta_{ij}X_{i}X_{j}, \end{array}$$

where *Y* is the predicted response, *β*_0_, *β*_*i*_, *β*_*ii*_, and *β*_*ij*_ are the regression coefficients for the intercept and the linear, quadratic, and interaction coefficients, respectively, *X*_*i*_ and *X*_*j*_ are the independent variables, and *k* = 2, i.e., the number of independent variables. The quality of the model fits was evaluated by the coefficients of determination (*R*^2^ and adjusted *R*_adj_^2^) and analysis of variance (ANOVA).

The levels of the tested variables and the observed response are shown in Table 2.

From the data for Cr(VI) adsorption in Table 2, a quadratic model was generated by RSM as it was statistically significant for the response variable. Analysis of variance (ANOVA) was applied for estimating the significance of the model at 5% significance level, and results are shown in Table 3. A model is considered significant if the *p* value (significance probability value) is less than 0.05. From the *p* values presented in Table 3, it can be stated that the linear terms *A* and *B* and quadratic terms *A*^2^ and *B*^2^ are significant model terms. The interaction term AB was not significant.

The model equation for uncoded (real) values of the quadratic model fitting experimental results is shown in the following equation:

(4) $$\begin{array}{c} \text{Cr}\left(VI\right)\text{ removal }\left(\%\right)\;=\;92.19\;+\;2.58A\;-\;5.74B\;-\;3.29A^{2}\;-\;9.65B^{2}. \end{array}$$

The coefficient of determination (*R*^2^) and the adjusted (*R*_adj_^2^) of the model were 0.951 and 0.939, respectively, which showed that this regression is statistically significant and only 4.90% of the total variations is not explained by the model.

**Figure 8:**
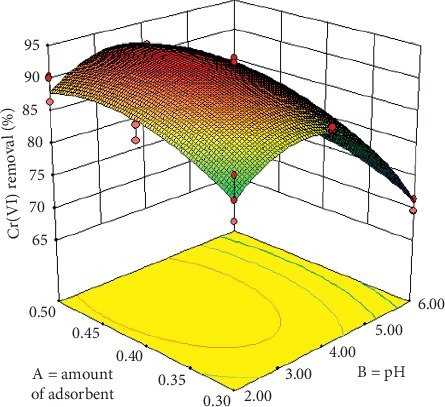
Response surface plot for Cr(VI) removal using organobentonite. Conditions: *C*_*i*_  = 50 mg/L, *V* = 50 mL, *T* = 20°C, stirring speed = 230 rpm, and contact time = 150 min.

Figure 8 shows the response surface plot that represents the interaction of the independent variables (amount of adsorbent and pH) on the removal of Cr(VI). In the pH range of 3.0 to 4.0, the removal of Cr(VI) is high and decreases at pH less than 2.5 or greater than 5.0. By means of mathematical optimization of the model, the values of the factors to which the maximum removal of Cr(VI) (93.16%) corresponding to pH of 3.4 and mass of adsorbent (Bent-HDTMA) of 0.44 g were determined.

Additional adsorption tests under the optimal operating conditions were carried out in order to evaluate the precision of the quadratic model. The experimental values and the predicted values with equation (4) are given in Table 4. Comparing the experimental and predicted results, it can be seen that the error between the experimental and predicted is less than 4.8%; therefore, it can be concluded that the generated model has sufficient accuracy to predict the Cr(VI) removal.

### 3.4. Adsorption Isotherms

**Figure 9:**
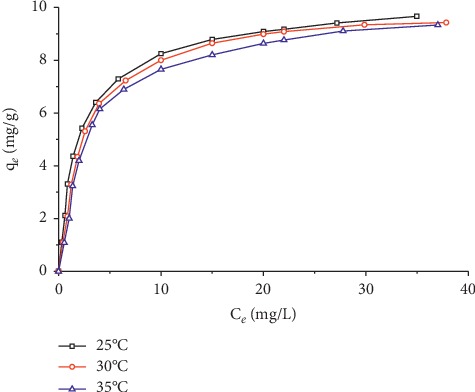
Adsorption isotherms for Cr(VI) removal onto organobentonite at different temperatures.

The Cr(VI) adsorption isotherms at 25, 30, and 35°C were evaluated at different initial concentrations of Cr(VI) ranging from 10 to 120 mg/L at the optimal conditions obtained from the experimental design, 0.44 g of organobentonite, and pH of 3.4. The other conditions were the same as those used in the experimental design (*V* = 50 mL, contact time = 150 min, and stirring speed = 230 rpm). The adsorption isotherms of Cr(VI) onto organobentonite are represented in Figure 9.

Adsorption isotherms data were fitted to the nonlinear form of Langmuir and Freundlich models, which are the most frequently used models [37]. The Langmuir isotherm is represented by the following equation:

(5) $$\begin{array}{c} q_{e}=\frac{Q_{o}bC_{e}}{1+bC_{e}}, \end{array}$$

where *q*_*e*_ is the adsorbate equilibrium amount in the solid phase (mg/g), *C*_*e*_ is the adsorbate equilibrium concentration in solution (mg/L), *Q*_*o*_ is the maximum adsorption capacity according to Langmuir monolayer adsorption (mg/g), and *b* is a constant according to the Langmuir model (L/mg). The characteristic of the Langmuir isotherm can also be expressed using a dimensionless constant called equilibrium parameter (*R*_*L*_), which has the following form:

(6) $$\begin{array}{c} R_{L}=\frac{1}{1+bC_{i}}, \end{array}$$

where *C*_*i*_ is the initial dye concentration in the solution (mg/L) and *R*_*L*_ value indicates the isotherm type, whether it is unfavorable (*R*_*L*_ > 1) , linear (*R*_*L*_=1), favorable (0 < *R*_*L*_ < 1), or irreversible (*R*_*L*_=0) [37].

The Freundlich isotherm model is an empirical relationship describing the adsorption of solutes from a liquid to a solid surface [37]. The form of the Freundlich equation is as follows:

(7) $$\begin{array}{c} q_{e}=K_{F}C_{e}^{1/n}, \end{array}$$

where *K*_*F*_ ((mg/g)(L/g)^*n*^) and *n* are the Freundlich constants related to the adsorption capacity and adsorption intensity of the adsorbent, respectively. Calculated isotherm parameters and correlation coefficients at the three temperatures are listed in Table 5.

Cr(VI) adsorption data onto organobentonite fit well with the Langmuir model with *R*^2^ greater than 0.993. The *Q*_*o*_ values, corresponding to the maximum adsorption capacity, were around 10.04 mg/g at 25°C, and the variation with the temperature was minimal. However, there is a slight decrease in the value of *q*_*e*_ with the temperature increase (Figure 8). Exothermicity of the adsorption results from the electrostatic interaction on the negatively charged Cr(VI) ions with the positively charged alkylammonium ions. The separation factor (*R*_*L*_) for the temperature range studied varied between 0.018 and 0.274, indicating the favorable nature of Cr(VI) adsorption on Bent-HDTMA, given that 0 < *R*_*L*_ < 1. The Freundlich model represented well the Cr(VI) adsorption data at low equilibrium concentrations, but deviated in the high concentration zone; hence, the coefficients of determination were less than 0.938. The values of 1/*n* in the range 0.1 < 1/*n* < 1 indicate that the adsorption is favorable.

Comparing with other low-cost adsorbents such as activated carbons prepared from coconut tree sawdust (3.46 mg/g at pH = 3.0) [38], mango kernel activated with H_3_PO_4_ (7.8 mg/g at pH = 2.0) [26], and groundnut husk (3.0 mg/g at pH = 3.0) [39], the adsorption capacity of the organobentonite synthetized in this study is higher (10.04 mg/g at pH = 3.4), indicating that this adsorbent has potential application for industrial wastewater treatment. The montmorillonite from Texas modified with octadecyltrimethylammonium bromide (9.61 mg/g at pH = 5.0) [40] and montmorillonite from Mongolia modified with hydroxyaluminium and cetyltrimethylammonium bromide simultaneously (7.64–9.09 mg/g at pH = 4.0) [25] presented *Q*_*o*_ values similar to those obtained in this study. However, the bentonite of San Luis Pososí (Mexico) modified with hexadecyltrimethylammonium bromide had a higher adsorption capacity (53.3 mg/g at pH = 3.0) [22], suggesting that the physicochemical and mineralogical characteristics of the starting clay and the organoclay preparation method affect the capacity of adsorption of Cr(VI).

## 4. Conclusions

In this study, the ability of organobentonite prepared from bentonite and a cationic surfactant (HDTMA-Br) to remove oxyanions of Cr(VI) in the aqueous solution was investigated. Response surface methodology (RSM) was used to optimize the conditions of Cr(VI) adsorption onto organobentonite, and the selected quadratic model can be used in predicting the Cr(VI) removal under the same conditions of the experiments. The optimal conditions for removal of Cr(VI) from an aqueous solution of 50 mg/L were pH of 3.4 and 0.44 g amount of the adsorbent. The percentage of Cr(VI) removal obtained was 93.16%. Stirring speeds above 200 rpm and contact times greater than 120 min did not have a significant effect on Cr(VI) removal. The error between predicted and experimental results for the removal of Cr(VI) is less than 4.8%, showing that the experimental results were in good agreement with those predicted from the quadratic model. Equilibrium data were well described by the Langmuir model with the monolayer adsorption capacity of 10.04 mg/g under optimal conditions at 25°C. The organobentonite can be a potential adsorbent to the remediation of water contaminated with chromium oxyanions, with the advantage of using as raw material for synthesis of the organoclay a natural, abundant, and low-cost mineral.

## Figures and Tables

**Table 1:** Experimental conditions for the initial adsorption tests of Cr(VI) in aqueous solution.

Test	pH	Stirring rate (rpm)	Adsorbent amount (g)	Contact time (min)	Ionic strength (added NaCl, mM)
1	1–9	200	0.2	60	Not controlled
2	3	100–300	0.2	60	Not controlled
3	3	200	0.05–0.40	60	Not controlled
4	3	200	0.2	30–240	Not controlled
5	3	200	0.2	60	0.1–2.0

**Table 2:** Factorial design for the independent variables used in this study along with the observed response.

Test	Code values		Real values		Response Cr(VI) removal, (%)
	A	B	A (mg)	B	
1	−1	−1	0.30	2.0	82.33 ± 3.16
2	0	−1	0.40	2.0	86.93 ± 1.28
3	1	−1	0.50	2.0	89.00 ± 2.15
4	−1	0	0.30	4.0	87.04 ± 0.25
5	0	0	0.40	4.0	92.28 ± 0.96
6	1	0	0.50	4.0	87.71 ± 5.63
7	−1	1	0.30	6.0	70.31 ± 1.11
8	0	1	0.40	6.0	78.07 ± 0.81
9	1	1	0.50	6.0	77.28 ± 4.54

**Table 3:** Results of regression analysis (ANOVA).

Source	Sum of square	DF	Mean square	*F* value	*p* value
Model	1337.65	5	267.52	80.60	<0.0001
A	119.38	1	119.38	35.97	<0.0001
B	592.70	1	592.70	178.57	<0.0001
AB	1.75	1	1.75	0.53	0.4755
AA	64.95	1	64.95	19.57	0.0002
BB	558.82	1	558.82	168.36	<0.0001

**Table 4:** Points for the validation of the experimental design.

Adsorbent amount (g)	pH	Cr(VI) removal, (%)		Error (%)
		Experimental	Calculated	
0.45	5.5	79.01 ± 0.83	82.78	4.78
0.35	3.0	91.17 ± 1.02	90.45	0.79
0.44	3.4	91.85 ± 0.92	93.60	1.75

**Table 5:** Equilibrium isotherm parameters for the adsorption of Cr(VI) onto organobentonite.

Model	25°C	30°C	35°C
Langmuir			
*Q*_*o*_ (mg/g)	10.04 ± 0.34	10.17 ± 0.29	10.04 ± 0.24
*b* (L/mg)	0.50 ± 0.06	0.38 ± 0.04	0.34 ± 0.02
*R*_*L*_	0.02–0.19	0.02–0.22	0.03–0.27
*R*^2^	0.998	0.997	0.993
Freundlich			
*K*_*F*_ (mg/g)(L/g)^*n*^	3.89 ± 0.43	3.33 ± 0.39	3.31 ± 0.37
*n*	3.68 ± 0.52	3.10 ± 0.43	3.17 ± 0.40
*R*^2^	0.938	0.936	0.923

## Data Availability

The data that support the characterization results of the organobentonite (XRD), the batch adsorption tests, and the complete factorial design data for optimization of Cr(VI) adsorption are found in the article in the form of tables and figures.
